# Successful Fetal Reduction in Early Second Trimester: Series of Three Cases Conceived With Infertility Treatment

**DOI:** 10.7759/cureus.54753

**Published:** 2024-02-23

**Authors:** Prachi A Ughade, Deepti Shrivastava

**Affiliations:** 1 Obstetrics and Gynecology, Jawaharlal Nehru Medical College, Datta Meghe Institute of Higher Education & Research, Wardha, IND

**Keywords:** cesarean section, ultrasound guidance, multiple pregnancies, assisted reproductive technologies, infertility treatment, fetal reduction

## Abstract

This case series explores three instances of a successful fetal reduction in early second-trimester pregnancies conceived through infertility treatments. The patients, all admitted to a central Indian tertiary care hospital, underwent assisted reproductive technologies such as in vitro fertilization (IVF) or intrauterine insemination (IUI). Faced with triplet pregnancies, fetal reduction was made to mitigate risks and enhance maternal and fetal well-being. The reduction procedures, conducted either transvaginally or transabdominally under continuous ultrasound guidance, resulted in the cessation of targeted fetal heart activity and motility. Post-reduction, pregnancies progressed without major complications, culminating in successful deliveries via lower segment cesarean section (LSCS) in the third trimester. This case series underscores the importance of selective fetal reduction in managing pregnancies arising from infertility treatments, emphasizing its role in minimizing risks associated with multiple gestations. Using continuous ultrasound guidance during the reduction procedures proved effective in ensuring precision and safety. These cases contribute valuable insights to the evolving field of reproductive medicine, offering clinicians a nuanced understanding of successful interventions to optimize outcomes in complex pregnancies.

## Introduction

Infertility treatments, such as in vitro fertilization (IVF) and intrauterine insemination (IUI), have revolutionized the field of reproductive medicine, offering hope to couples facing challenges in conceiving naturally. As these interventions become more prevalent, the occurrence of multiple pregnancies following assisted reproductive technologies (ART) raises significant clinical concerns [1]. Multiple gestations are associated with increased risks of maternal and fetal complications, including preterm birth, low birth weight, and gestational hypertension [1,2]. Successful fetal reduction in early second-trimester multifetal pregnancies can be accomplished through selective termination, a procedure aimed at reducing the number of fetuses in multiple pregnancies. Technical success rates for this technique have been reported at 100%, with miscarriage rates varying at different gestational stages: 12.6% before 24 weeks, 5.4% when performed at less than or equal to 16 weeks, and 14.4% when done thereafter [3]. Selective multifetal pregnancy reduction in the second trimester offers advantages such as objectively addressing abnormalities in the fetus or effectively reducing the fetal number [4]. This procedure is considered safe and decreases the complications associated with multifetal pregnancies while increasing the overall birth rate [4].

Selective fetal termination is a medical procedure performed to reduce the number of fetuses in a multiple pregnancy while ensuring the health of the remaining fetuses. It can be carried out through surgical, medical, or labor induction methods, depending on factors like gestational age and maternal health. Patients considering this procedure must receive comprehensive counseling and provide informed consent. Legal and ethical considerations are essential, and adherence to applicable laws and guidelines is necessary. Overall, selective fetal termination aims to optimize outcomes in complex pregnancies through careful assessment and individualized decision-making [4]. When the objective is to reduce the number of fetuses, the fetus in the fundus uteri is typically chosen, reducing the multifetal pregnancy to twins [4]. It is recommended to refrain from performing the procedure during instances of vaginal bleeding, opting to reduce the fetus only after vaginal bleeding has ceased for one or more weeks [4]. However, it is crucial to acknowledge potential risks and considerations associated with multifetal pregnancy reduction, including coagulopathy or ischemic damage in survivors [3]. Patients should receive comprehensive counseling that includes the latest information on the abnormality or disease, empowering them to make informed decisions tailored to their unique circumstances [4]. Furthermore, it is imperative to recognize that state and federal laws may influence the provision of selective reduction [5]. Fetal reduction, a procedure aimed at reducing the number of fetuses in multiple pregnancies, has emerged as a viable strategy to manage these risks and improve overall outcomes. This case series presents three instances of successful fetal reduction in the early second trimester, focusing on pregnancies conceived through infertility treatments. The cases underscore the importance of timely and well-guided reduction procedures in minimizing potential complications and optimizing the health of both mother and infants.

The decision to undergo fetal reduction is a complex one, necessitating careful consideration of factors such as the number of fetuses, maternal health, and the gestational age at diagnosis. The procedures performed either transvaginally or transabdominally under continuous ultrasound guidance aim to selectively reduce the number of fetuses while preserving the health and development of the remaining ones [6]. This series contributes to the growing body of literature on the management of multiple pregnancies following infertility treatments, shedding light on the efficacy and safety of selective fetal reduction. The outcomes presented in these cases provide valuable insights for clinicians involved in the care of women undergoing ART, emphasizing the importance of personalized and evidence-based approaches to optimize maternal and fetal well-being.

## Case presentation

Case one

**Figure 1:**
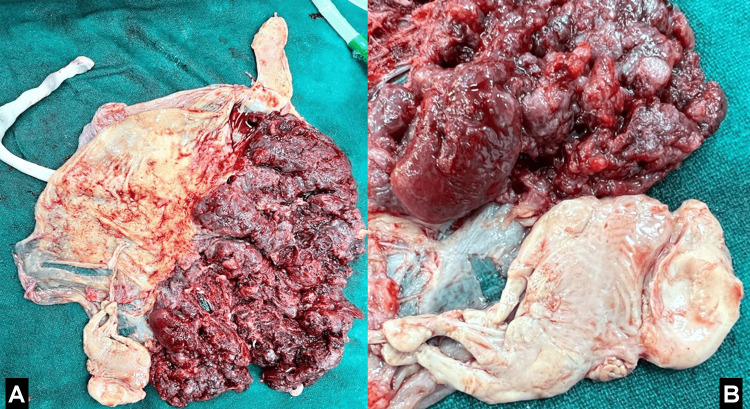
(A) Placenta with cotyledons intact. (B) Reduced fetus of 13-week gestational age.

A 28-year-old primigravida woman was admitted to a tertiary care hospital in central India in July 2023, having conceived through IVF. She presented with a diminished ovarian reserve, indicated by an anti-Müllerian hormone (AMH) level of 0.2 and an antral follicle count (AFC) of 3. She underwent a fresh embryo transfer with three to three embryos as part of a two-cycle IVF package. The ovum pick-up procedure was initially performed, followed by embryo transfer a few days later. The first ultrasound revealed a triplet pregnancy. After obtaining consent, a decision was made to reduce the number of fetuses to twins at 13 weeks, considering the risk of early pregnancy losses. The selective fetal reduction was carried out transvaginally, and the pregnancy progressed without complications. After the delivery, the placenta with a reduced fetus is shown in Figures 1A, 1B. At 38 weeks into her pregnancy, during the third stage, the patient reported leaking per vagina and lower abdominal discomfort, prompting an urgent lower segment cesarean section (LSCS) for twin pregnancy. The first twin weighed 2.1 kg at birth, while the second weighed 2 kg. The APGAR scores for the first twin were 8/10 at 5 minutes and 10/10 at 10 minutes, and for the second twin, they were 8/10 at 5 minutes and 9/10 at 10 minutes. Both babies were then placed by the mother's side.

Case two

**Figure 2:**
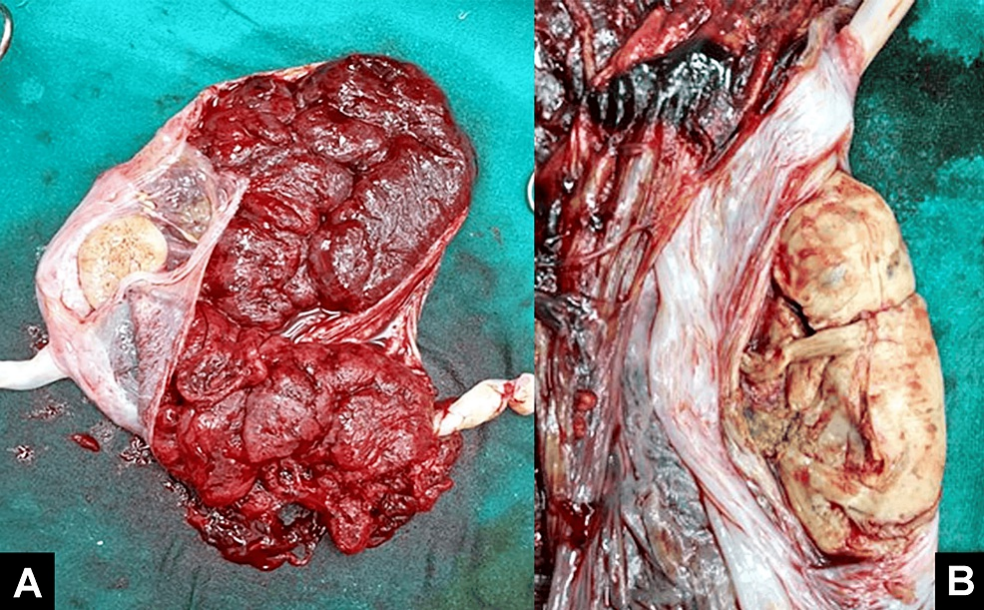
(A) Placenta with cotyledons intact. (B) Reduced fetus of 13-week gestational age.

A 35-year-old primigravida female was admitted to a tertiary care hospital in central India in September 2022, having undergone IVF. She had a history of unsuccessful frozen embryo transfers. With an AMH value of 2.1, she was chosen for a frozen embryo transfer using three day-two embryos as part of a three-cycle IVF package. The ovum pick-up procedure was initially performed, followed by embryo transfer after a few days. The first ultrasound revealed a triplet pregnancy, leading to a decision to reduce the fetuses to twins at 14 weeks, considering the risk of early pregnancy losses. The transabdominal selective fetal reduction was performed after obtaining consent, and the pregnancy progressed without complications. After the delivery placenta with a reduced fetus is shown in Figures 2A, 2B. In the third trimester, at 38 weeks of pregnancy, the patient reported lower abdominal pain, prompting an emergency LSCS for malpresentation. The first twin weighed 2 kg at birth, while the second weighed 1.9 kg. The APGAR scores for the first twin were 8/10 at five minutes and 10/10 at 10 minutes, and for the second twin, they were 8/10 at five minutes and 9/10 at 10 minutes. Both infants were then placed by the mother's side.

Case three

**Figure 3:**
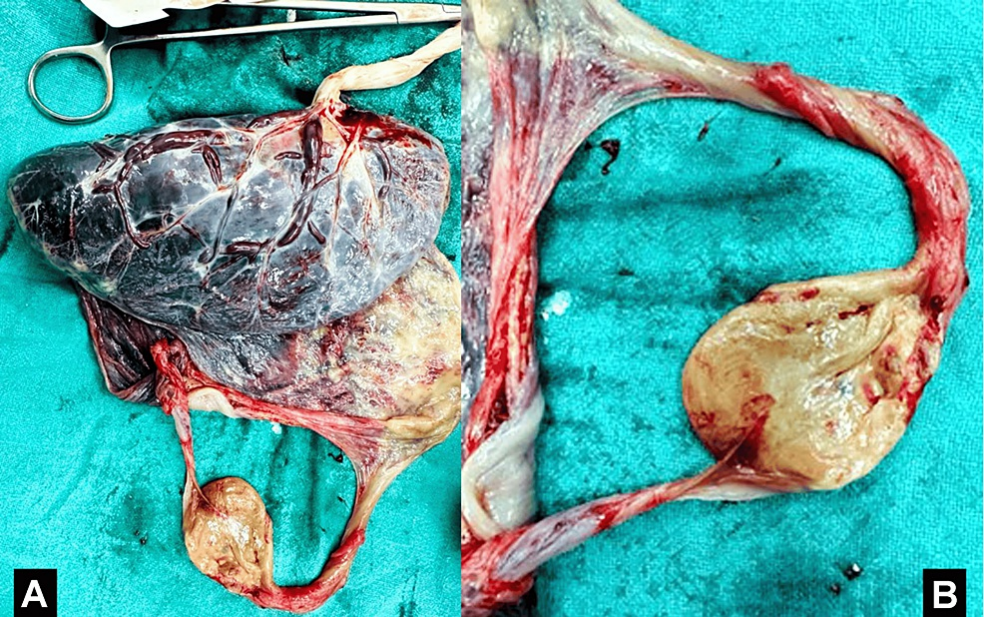
(A) Placenta with cotyledons intact. (B) Reduced fetus of 13-week gestational age.

A 36-year-old woman was admitted to a tertiary care hospital in central India in October 2022, who had previously undergone three IUI cycles with letrozole tablets and human chorionic gonadotropin (HCG) injections without success, was admitted to a tertiary care hospital in central India. This time, she underwent an IUI cycle using clomiphene citrate tablets, human menopausal gonadotropin injections, and HCG injections, resulting in a successful conception. Upon her first ultrasound, a twin pregnancy was diagnosed. With consideration for the mother's hypertensive condition and to mitigate risks and improve both maternal and fetal outcomes, a decision was made, with consent, to reduce the number of fetuses to a single at 14 weeks. A transabdominal procedure was employed for selective fetal reduction. After the delivery, placenta with a reduced fetus is shown in Figures 3A, 3B.

In the third trimester, at 38 weeks of pregnancy, the patient experienced lower abdominal pain, leading to an emergency LSCS due to a non-reassuring cardiotocography (CTG). The newborn girl had a birth weight of 2.7 kg, and her APGAR score was 8/10 at 5 minutes and 9/10 at 10 minutes. The baby was subsequently shifted to the mother's side.

Under the guidance of ultrasound (USG), a selective fetal reduction procedure was conducted either transvaginally or transabdominally. A 15 cm, 22-gauge needle was utilized with strict aseptic precautions to access the fetal thoracic cavity, aiming to reduce the fetus under continuous ultrasound guidance. The procedure resulted in the cessation of fetal heart activity and motility in the targeted fetus. For the reduction of the Injection KCL was used after the reduction, the remaining fetuses displayed normal heartbeats and movements. After three hours, a confirmation scan was performed, affirming the absence of cardiac activity in the reduced fetus and the continued cardiac activity in the unaffected fetuses. The patient was advised to rest for 24 hours. A second scan conducted at 22 weeks of gestation revealed normal findings.

## Discussion

Infertility treatments, particularly IVF and IUI, have become increasingly prevalent methods for achieving pregnancy in couples facing fertility challenges. However, the elevated incidence of multiple pregnancies associated with these interventions brings forth concerns about maternal and neonatal well-being. The cases in this series demonstrate the proactive approach of clinicians in addressing these concerns through timely and well-planned fetal reduction procedures. Various factors, such as maternal age, obstetric history, and underlying medical conditions, influenced the decision-making process for fetal reduction in each case. Notably, the reduction procedures were performed at different gestational ages, ranging from 13 to 14 weeks, reflecting these decisions' individualized and patient-centric nature. This personalized approach aligns with the recommendations outlined in previous studies emphasizing the importance of considering maternal and fetal factors when determining the optimal timing for fetal reduction [7,8].

Continuous ultrasound guidance during the reduction procedures was crucial in ensuring precision and safety. Using a 15 cm, 22-gauge needle for access to the fetal thoracic cavity, as described in the methods, conforms to established protocols for these interventions [9]. The confirmation scans post-reduction, coupled with subsequent follow-up ultrasounds, provided a comprehensive assessment of fetal well-being and confirmed the success of the reduction while ensuring the health of the remaining fetuses. The three cases presented in this series demonstrate favorable neonatal outcomes, with infants born at term via LSCS exhibiting encouraging APGAR scores. These outcomes align with previous studies emphasizing the positive impact of fetal reduction on perinatal outcomes in multiple pregnancies [10,11]. While the series highlights successful cases, it is essential to acknowledge that the decision for fetal reduction is complex and involves ethical considerations. Shared decision-making, thorough counseling, and obtaining informed consent are integral components of the process, as reflected in the detailed descriptions of the cases. Patient education regarding fetal reduction's potential risks and benefits is paramount to ensure that individuals participate actively in the decision-making process.

## Conclusions

In conclusion, this case series provides valuable insights into the successful implementation of selective fetal reduction in pregnancies conceived through infertility treatments, emphasizing the importance of personalized care and continuous ultrasound guidance. The decision to undergo fetal reduction, driven by concerns for maternal and fetal well-being, reflects the evolving landscape of reproductive medicine. The favorable neonatal outcomes observed in the presented cases, including term deliveries and encouraging APGAR scores, underscore the potential benefits of timely and well-planned fetal reduction in mitigating risks associated with multiple pregnancies. The cases also highlight the adaptability of reduction procedures, either transvaginal or transabdominal, in addressing each patient's unique needs. Continuous ultrasound guidance emerged as a crucial tool, ensuring precision and safety during the reduction procedures. The meticulous follow-up, including confirmation scans and subsequent ultrasounds, further affirmed the success of the reduction while providing ongoing reassurance regarding the health of the remaining fetuses. While the series demonstrates positive outcomes, it is essential to acknowledge the complexity of the decision-making process surrounding fetal reduction. Ethical considerations, shared decision-making, and comprehensive patient counseling are integral components of this process, emphasizing the importance of patient autonomy and informed consent. Looking forward, the presented cases contribute to the growing body of evidence supporting the efficacy and safety of selective fetal reduction in the context of infertility treatments. Larger-scale studies and long-term follow-ups are warranted to provide additional insights into these interventions' broader implications and outcomes.
